# Mesenchymal Stem Cells: A Trump Card for the Treatment of Diabetes?

**DOI:** 10.3390/biomedicines8050112

**Published:** 2020-05-06

**Authors:** Elisabetta Donzelli, Arianna Scuteri

**Affiliations:** Experimental Neurology Unit and Milan Center for Neuroscience, School of Medicine and Surgery, University of Milano-Bicocca, Via Cadore 48, 20900 Monza, Italy; elisabetta.donzelli@unimib.it

**Keywords:** type-1 diabetes, type-2 diabetes, mesenchymal stem cells, pancreatic islets transplantation, insulin, immune suppression

## Abstract

The advent of the new revolutionary approach based on regenerative medicine is progressively reshaping the therapeutic scenario of many different diseases, such as cardiovascular diseases and immune diseases, with encouraging results. During the last 10 years, many studies have also proposed the use of mesenchymal stem cells (MSCs), adult stem cells with several interesting properties already used in different experimental models, for the treatment of diabetes, however, reporting conflicting outcomes. These reasons have given rise to a question: are these cells a real trump card for the biomedical field? Are they really able to outclass the traditional therapies, or at least able to give an advantage over them? In this review, we will discuss the most promising results obtained with MSCs for the treatment of diabetes and its complications, we will compare the different therapeutic treatments applied as well as the most likely mechanisms of action, and overall we will give an in-depth overview of the pros and the cons of the use of MSCs for the therapy of both type-1 and type-2 diabetes.

## 1. Introduction

The term “diabetes” as a matter of fact groups together a series of several pathologies, which differ one from the other for etiology and incidence, but which all have the condition hyperglycemia in common, as stated by the American Diabetes Association (ADA) [1]. This condition may be due to a defective insulin production, or arising as a consequence of reduced insulin responsiveness in peripheral tissues. Insulin produced by beta cells of pancreatic islets is essential to allow the entry of glucose into cells and therefore its use as cellular “fuel”. Without insulin, cells are no longer able to exploit glucose for ATP production, necessary for their correct function and survival. As a consequence, alternative fuel molecules must be used by the body, such as fatty acids, with the final production of ketone bodies and the onset of ketoacidosis, a life-threatening complication of diabetes. On the contrary, glucose remains in vessels, where it may cause several severe damages due to protein glycation.

Clinically, nowadays at least five different forms of “diabetes” have been identified, but type-1 diabetes (T1D) and type-2 diabetes (T2D) are the most predominant ones. As a general assumption, T1D is considered as a juvenile disease, while T2D is considered a pathology of elderly people. However, the differences rely more on the pathogenic mechanisms than on the age of onset. Moreover, in T2D, hyperglycemia derives from a relevant peripheral insulin resistance and this is the form with the highest incidence, accounting for nearly 85%–90% of cases; it often occurs in the elderly, but it can also arise in overweight or obese young people. On the other hand, T1D is an autoimmune disease with a prevalent but not exclusive juvenile onset; it is caused by the production of self-antibodies that progressively destroy pancreatic beta cells, responsible for insulin production. T1D affects about 15% of patients [2]. Being the most common diabetes forms, the research mainly focused on the study of T1D and T2D.

## 2. Diabetes Treatment

### 2.1. Exogenous Insulin Administration

**Figure 1:**
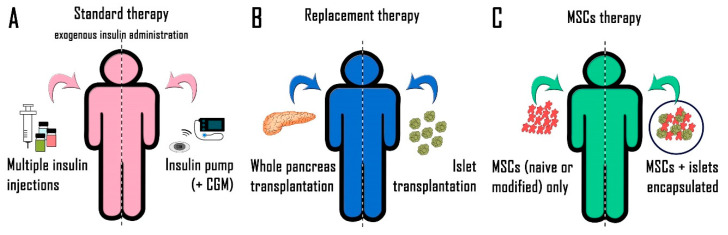
A schematic representation of current or proposed therapies for diabetes. (**A**) The standard therapy for diabetes, based on exogenous insulin administration by multiple injections or insulin pump. (**B**) The therapies based on the replacement of pancreatic islets, either by whole pancreas transplantation or by isolated islet transplantation. (**C**) The new therapies based on MSCs (mesenchymal stem cells). Given alone or encapsulated into 3D structures together with pancreatic islets.

Diabetes is an ancient disease, since it is possible to find a description of its peculiar features and symptoms even in Egyptian papers. The name itself, “diabetes”, was coined by Aretaeus of Cappadocia to describe a disease which makes the body act as a siphon (precisely diabetes in Greek), to eliminate the water [3]. The correlation of diabetes with the pancreas has only been understood since 1889, but it was only in 1922 that the disease, lethal until that point, found its first treatment, that is, exogenous insulin administration, thanks to Banting and Macleod, an overall and effective setting up of insulin extraction [3]. The history of diabetes treatment started in this way and has been refined during the years, although no substantial changes have been introduced during almost a century from the first treatment. Insulin is now synthesized and no longer extracted. New insulin profiles have been developed with different rates of release (fast or slow) and innovative administration devices have been introduced, such as micro infusion pumps [4], but the administration of exogenous insulin largely still remains the diabetes gold standard treatment in particular (but not exclusively) for T1D. The rationale behind the therapy is very simple: insulin level is too low, or at least it is insufficient, therefore it must be administered to the patient (see Figure 1A). The treatment is effective, and it is quite simple to be performed, but such a therapy also presents some limits and side effects. In fact, in physiological healthy conditions, pancreatic islets beta cells immediately react to blood glucose level variations by a real time release of endogenous insulin in order to maintain glycemia at nearly constant levels. On the contrary, in diabetic patients, exogenous insulin is administered as a bolus at different times during the day (or when it is needed) after a glycemia check. This kind of administration however results in a series of peaks, both of hyperglycemia and of hypoglycemia, without a constant and harmonic regulation of the blood glucose level. Even if the situation has been improved with respect to the last century by the formulation of insulins with different kinetic profiles and by the introduction of insulin pumps (i.e., devices for semi-automatic insulin subcutaneous administration, nowadays often combined with continuous glucose monitoring (CGM) systems [4]), the constant control of blood glucose level still remains a utopian goal. Moreover, the alternation of hyper and hypoglycemia peaks exposes the body to cyclically high or dangerously low levels of blood glucose, with important severe consequences also at the microcirculation, thus increasing the risk of developing the main long-term side effects of diabetes, such as nephropathy and neuropathy [4,5]. Such side effects drastically affect the patient’s quality of life, and they could also lead to lethal consequences. Moreover, it is noteworthy that the patients have to administer the therapy by themselves, and therefore the effectiveness of the treatment is strictly dependent on correct compliance. Without the constant and active collaboration and participation of the patient, the therapy effectiveness drastically falls. For all these reasons, during the years the research for new treatments, alternative to insulin administration, to manage diabetes has never stopped.

### 2.2. New Therapeutic Approaches

#### 2.2.1. Whole Pancreas Transplantation

A pioneering approach has been attempted at the end of sixties by a team from the University of Minnesota, who performed the first whole pancreas transplantation [3] (See Figure 1B). Once again, the rationale was simple: a pancreas component (beta cells) does not work, so it would be completely replaced by complete pancreas transplantation. Theoretically, it was the ideal approach, able to restore with a single intervention the physiological glycemia control and it produced encouraging results. However, from a practical point of view, this approach presents a number of problematic variables since it is very invasive for the patient and, as in all organ transplantation, it needs a compatible donor. Even more relevant, it requires the establishment of a lifelong immune-suppressive treatment after the transplantation, with severe side effects. For these reasons, although effective, a similar approach could not replace the simple therapy based on insulin administration due to its side effects and its very limited feasibility [6].

#### 2.2.2. Pancreatic Islet Transplantation

A more significant approach was developed as a further evolution of whole pancreas transplantation, represented by pancreatic islets transplantation. In this case, the patient receives just pancreatic islets that are the endocrine part of the pancreas responsible for insulin secretion based on blood glucose levels (see Figure 1B). Such an approach is far less invasive for the patient since it is not necessary to transplant pancreatic islets into the pancreas. They could be transplanted into any organ vascularized enough to sustain their survival, typically into the liver or under the renal capsule, and they can ensure a physiological control of blood glucose level. Unfortunately, this approach presents important negative aspects: (i) a high number of pancreatic islets, derived from more than a cadaveric donor (4–5 donors may be necessary), is required to achieve the correct glycemic control [7]; (ii) the immune-suppressive therapy is mandatory even in this setting; and overall (iii) the transplanted tissue has a limited duration (also caused by pericapsular fibrosis, as reported by Vaithilingam et al. [8]), so that multiple transplantations are necessary during the patient’s life. On the basis of such considerations, it would be extremely difficult for pancreatic islet transplantation to replace insulin administration as the standard therapy for diabetic patients.

In this scenario, a very promising and revolutionary step forward is represented by regenerative medicine, and more details of the possibility of obtaining beta-like cells derived from stem cells.

#### 2.2.3. Beta-Cell Replacement

The discovery of the great differentiation potential of stem cells started several years ago with the ambitious aim of obtaining pancreatic beta cells derived from stem cells. In particular, recent progresses have been made by using human pluripotent stem cells (hPSCs), including human embryonic stem cells (hESCs) and human induced pluripotent stem cells (hi-PSCs) [9]. Many authors have tried to force these cells to undergo a specific differentiation by using cocktails of growth factors or drugs to inhibit or to activate precise signaling pathways, such as TGF-β signaling pathway [10,11], or by driving the expression of genes peculiar for beta cells though genetic manipulations [12]. The greater part of these studies was able to obtain a limited amount of cells which expressed beta cell markers and responded to glucose stimulation. Unfortunately, these cells were not able to release an insulin amount comparable to that released by pancreatic islets beta cells, therefore not being functional. In addition, embryonic stem cells also presented an increased risk of tumorigenesis and immune rejection [13,14]. The latter problem could be bypassed by using hi-PSCs, which could be derived directly from the patient who will receive them after in vitro manipulation. Moreover, the new gene editing techniques, such as CRISPR/Cas9, make it possible to correct the genetic defects responsible for diabetes, as well as to introduce pro-differentiative genes [12]. Therefore, hi-PSCs seem to be more promising as a therapeutic option and more complex and effective protocols have been recently proposed focused on these cells, such as the multi-steps protocol by Velazco-Cruz (2019), which was able to obtain an almost pure and functional population of beta-like cells though the time-dependent modulation of TGF-β signaling pathway [15]. Furthermore, although cell replacement represents a very intriguing therapeutic option for the future, different problems remain to be solved, such as the identification of the best transplantation method as well as the high costs of differentiation protocols, their complexity and their related risks [13,16]. For these reasons, other alternative options have been explored, and besides differentiation, in the last years it has become more and more evident that other stem cells, such as mesenchymal stem cells (MSCs), possess interesting properties which could be exploited for the treatment of diabetes [17].

## 3. Mesenchymal Stem Cells

MSCs are adult stem cells resident in different tissues, such as bone marrow, adipose tissue and perinatal tissues (umbilical cord, Wharton’s jelly) and are easily harvested and expanded. At first, these cells also grabbed the attention of the scientific community for their potential ability to replace damaged organs and cells, but then they realized that the protective positive action of such cells could be focused on other MSC properties, different from their differentiation potential. In particular, MSCs are able to home towards a lesion site, they are able to release a huge number of soluble factors and, overall, they are hypoimmunogenic and possess important immunomodulatory properties. Despite the downfall of the transdifferentiation hypothesis, many authors have demonstrated that MSCs are able to acquire an insulin-releasing phenotype [18].

MSCs have been proposed for the treatment of both T1D and T2D and they have been studied in many different experimental models, being potentially useful in a dual way: (i) administered on their own, or (ii) partnered with pancreatic islets, with the aim in both the cases being to exploit their manifold properties (See Figure 1C). Very promising results have been achieved with both the administration models and, on the other hand, both ways present potential pitfalls and limits. In this review, we will discuss the most promising results obtained with MSCs for the treatment of diabetes and its complications; we will compare the different therapeutic treatments applied as well as the most likely mechanisms of action and overall we will give an in-depth overview of the pros and the cons of the use of MSCs for the therapy of both T1D and T2D [17,19,20].

### 3.1. Administration of MSCs Alone

On a theoretical basis, each ability of MSCs could be exploited to counteract the events or conditions that lead to the development of diabetes or its long-term effects: immune-modulation may be used to reduce autoimmune destruction of pancreatic beta cells, as well as the inflammatory state related to microvascular alterations involved in long-term consequences. Moreover, MSCs could stimulate the endogen beta cells regeneration, as well as support their survival. As a consequence, both T1D and T2D may benefit from the use of MSCs [17,21].

These were the starting points which motivated further studies in order to shed light on MSCs potential therapeutic action for diabetes treatment and, overall, to verify if MSCs’ abilities were still effective upon in vivo transplantation.

During the last years many studies have evaluated the effectiveness of MSCs transplanted on their own, in different experimental models, different ways of administration and, overall, with different and sometimes contrasting results. 

#### 3.1.1. In Vitro Studies

Most papers agree on the positive effect of MSCs on diabetes in in vitro experimental models. In particular, it has been demonstrated that these cells are able to support both survival and function of pancreatic islets, by different mechanisms of action: while the direct contact promoted the differentiation of MSCs into an insulin-releasing phenotype, their paracrine action supported the islet survival [18]. Concerning the effect on islet survival, the analysis of MSCs secretoma revealed that these cells release a plethora of soluble factors with a trophic action on pancreatic beta cells, as well as a number of anti-inflammatory cytokines, antioxidant factors and anti-apoptotic molecules, which could theoretically relieve the inflammatory milieu favorable to diabetes onset and to insulin resistance development [22]. Besides such paracrine effects on pancreatic islets, MSCs could also exert a positive action through direct contact with pancreatic islets: after this mutual interaction, they can start to release insulin, thus becoming insulin producing cells [18,20]. Many mechanisms may be involved in such a direct stimulation between MSCs and pancreatic islets, most of them are still unknown, although it has been proposed an important role of the extracellular matrix proteins [23], as well as of the exosomes (and more generally extracellular vesicles) exchange. Exosomes are small-membrane vesicles with a size of 40 to 150 nm which are emerging as pivotal for cell-to-cell communication, being responsible for a horizontal transfer (i.e., mediated by a cell-to-cell exosome exchange) of proteins [24]. The exosome content is dynamically arranged and it depends on MSCs’ origin as well as on MSCs’ intercellular connections, ranging from regulatory miRNAs, anti-inflammatory molecules, trophic factors and even subcellular components [25]. All these factors could play a role in the MSC positive effect observed in vitro on pancreatic islets.

#### 3.1.2. In Vivo Studies

In vivo, the situation may be more complex. Many authors have observed a positive effect of MSCs’ administration [23,24,26,27,28]. By analyzing the single steps involved in diabetes onset, MSCs have also demonstrated being able to specifically modulate hyper-reactive T cells, those responsible for pancreatic beta cells destruction, without compromising the whole body’s immunity [23]. Such effect was mainly achieved by the release of soluble factors, such as hepatocyte growth factor (HGF), Il-10 and Il-6, which concurred to modify the pancreatic micro environment and to counteract the disease. In fact, diabetes progression is generally related to the existence of a pro-inflammatory condition, made up by the pro-inflammatory cytokines released by lymphocytes and macrophages. Additionally, there exists a fine balance between the anti-inflammatory cytokines released by Th-2 lymphocytes and the pro-inflammatory ones released by Th-1 lymphocytes [24]. The same balance exists between anti-inflammatory M2 and pro-inflammatory M1 macrophages [26,27]. The administration of MSCs in in vivo models of diabetes was reported to induce an increase in the number of M2 macrophages as well as of Th-2 lymphocytes, thus switching the balance towards the anti-inflammatory cytokines [15,17,18]. In this way, the inflammatory state which evoked beta cell destruction and insulin resistance was replaced by a milieu promoting pancreatic islets endogen regeneration.

Besides the immune-modulatory effect, MSCs also released some molecules (in particular annexin 1, stromal-cell derived factor 1 (SDF-1) and complement component 3), which act as ligands for the G-protein coupled receptor (GPCR) present on islets. When administered to pancreatic islets, on their own or in combination, such molecules were able to support the islets’ survival, as well as to increase the glucose secretion [28].

Although all this encouraging evidence, the in vivo effectiveness of MSCs administered on their own to control glycemia in the diabetes treatment is not so simple to be exploited: some authors observed that MSCs alone were unable to in diabetic animals, at least after a single injection [29,30], and generally they reported contrasting results [31]. In addition, even if MSCs derived from different organs could have a positive action [32], there are important differences related to tissue derivation in terms of effectiveness rate, due to their extreme variability (in particular for human MSCs), with better outcomes achieved reported with perinatal tissue-derived MSCs [19]. In Table 1, we reported a list of the different sources of MSCs used in the papers cited in the present review, which diabetes type they were addressed to and the more likely mechanism of action suggested.

#### 3.1.3. MSC Manipulation

To bypass the MSCs’ heterogeneity and to make them a more confident tool, some authors suggested manipulating MSCs in order to enhance their insulin release and their pro-regenerative properties [33,38,39]. Different kinds of manipulation have been investigated, and the most used and simple strategy relies on the “activation” of MSCs, which is generally obtained by a previous exposition to hypoxia. Different authors demonstrated that, in such hypoxic conditions, MSCs increased their proliferation rate, as well as the release of pro-survival and anti-apoptotic molecules, such as VEGF, Il-6 and Akt [38,40]. In such an “activated state”, MSCs were able to improve their stimulation of tissue repair and their immunomodulation properties [19]. Another important strategy to improve MSCs’ performance is based on genetic manipulation, mainly by transfection with viral vectors, with the aim to increase the expression of those genes pivotal for insulin production/glucose uptake and islet survival, such as Pdx-1, apelin, and VEGF [19,33,39]. Although all these techniques seem to be promising and useful to enhance the positive trophic action of MSCs for diabetes treatment, their main limit is represented by the potential risks related both to the use of viral vectors and, more generally, to cell manipulation which can lead to uncontrolled and unpredictable effects. A method to bypass such limitation is represented by the new frontier of cell-free based therapy [25]. Different authors have demonstrated that the same positive effect obtained with MSCs in diabetes experimental models could be achieved also by using MSC-derived exosomes [21,34,37]. Repeated doses of MSC-derived exosomes administered i.v. in diabetic animals were able to increase insulin production and to reduce pancreatic beta cell degeneration, also acting at molecular level by increasing the expression of glucose transporter 4 (GT-4), thus accelerating glucose metabolism [37]. Although results are very promising, the control of glycemia obtained with MSCs treatment remains transient, and the pancreatic beta cell regeneration is still far from being effective in reversing the disease. Further and detailed analyses are required to hypothesize the use of MSCs as a unique treatment to counteract diabetes effects. Nevertheless, there is another intriguing therapeutic approach which relies on the use of MSCs and their peculiar properties: the co-transplantation of MSCs with pancreatic islets. 

### 3.2. Co-Transplantation of MSCs and Pancreatic Islets

As stated before, the transplantation of pancreatic islets is a new therapeutic option, alternative to the classical administration of exogenous insulin, whose clinical use is limited mainly by the shortage of transplanted tissue. The benefit provided by MSCs would be firstly represented by an increase in islet survival, and eventually by an enhancement of islet function. Therefore, the therapeutic rationale is not aimed to counteract the pathogenic mechanisms causing diabetes, but to strengthen the outcome of pancreatic islet transplantation, thus improving its clinical feasibility. As a matter of fact, all the peculiar features of MSCs mentioned above could be theoretically useful to prolong pancreatic islet survival, or to protect them from the immune-mediated attack. Many authors have explored the effect of MSCs, both naïve and manipulated (as previously described), co-transplanted with pancreatic islets in in vitro and in vivo studies. 

#### 3.2.1. Role of Soluble Factors 

All the studies evidenced a better outcome for pancreatic islets transplantation in the presence of MSCs, with a lower number of islets necessary to reach the glycemic stabilization and a higher duration of symptoms control. Islets showed a better vascularization, ascribable to the angiogenic properties of MSCs (already observed when transplanted on their own), through the release of VEGF. The soluble factor increased the blood supply to islets, thus on one hand making them more exposed to glucose level variations and improving their performance, and on the other hand, it allowed a greater amount of nutrients and oxygen to reach the islets, thus increasing their survival. VEGF was also reported to activate the pro-survival pathway PI-3K/Akt/m-TOR, thus improving islet lifespan [41]. In addition, many authors evidenced a reduction of oxidative stress and inflammation, which are among the main causes responsible for transplanted islets shortage. This observation underlines once again the important role of soluble factors, together with the evidence that, when administered i.v., MSCs are often trapped at lung level and do not reach pancreatic islets, thus supporting the paracrine action [42]. In this context, MSCs could also be hypothesized as trophic factors carriers, also considering the option to engineer them to force the expression of relevant genes [43]. However, the release of soluble factors is not the only mechanism proposed to explain the positive effect of MSCs. 

#### 3.2.2. Role of Direct Contact

Montanari et al. [35] underlined the importance of direct contact too: they observed an increased expression of adhesion molecules ICAM-1 and N-cadherin. When these molecules were neutralized by specific antibodies, the positive effect of MSCs disappeared. Even more interesting, the direct contact between MSCs and pancreatic islets seemed to further enhance the anti-diabetes MSCs’ effect as well as their positive action on pancreatic islets, thus improving the transplantation outcome [36]. Arzouni and colleagues [44] reported that MSCs were able to enhance insulin production by pancreatic islets, and such an effect was stronger when MSCs were previously exposed to inflammatory cytokines, which switched MSCs into an anti-inflammatory phenotype. The direct contact between MSCs and pancreatic islets is thought to trigger the differentiation into insulin-producing cells, thus directly contributing to increasing the insulin amount and improving islets’ function. However, in vivo, it is not so simple to maintain the direct contact between pancreatic islets and MSCs, as it occurs in in vitro co-culture. To also guarantee the direct contact between MSCs and pancreatic islets when transplanted into the living organism, a technique has been set up for their co-encapsulation into a 3D matrix of inert materials, such as alginate. Such encapsulation also allowed the masking and hiding of pancreatic islets from the immune system, thus making it no longer necessary to use immunosuppressive drugs to prevent immune rejection against donor-derived pancreatic islets. Another method introduced by Borg and colleagues [45] is based on a 3D co-culture system with a cryogel scaffold of PEG and heparin. Such a device allowed the co-housing of pancreatic islets and MSCs and their interactions.

The results obtained with MSCs co-administered with pancreatic islets are encouraging and they created more general agreement and less discordant reports with respect to the results obtained with the MSCs-only injection. Furthermore, in these cases the control of blood glucose level was also revealed to be transient and, after a period of variable time and different for each individual, the requirement of exogenous insulin administration appeared again.

### 3.3. Clinical Use of MSCs

All these promising results, obtained both with MSCs alone and with MSCs partnered with pancreatic islets, have represented the starting point which has allowed the design, development and registering of approximately 70 different clinical trials, both for T1D and for T2D (see clinicaltrials.gov), based on MSCs’ administration. So far, the clinical safety of the infusion of both autologous and allogenic MSCs has been demonstrated. Many trials also reported the effectiveness of MSCs’ treatment by exploring very different administration protocols and in different patients. In different clinical settings, the effect of MSCs’ administration was evaluated in patients with different histories of diabetes (both T1D and T2D): some studies took into account the MSCs’ action in patients with a very recent diagnosis (less than three weeks before the enrollment) [46], while other ones were focused on patients with an older diagnosis. In all the studies, MSCs were able in most cases, to improve diabetes symptoms and reducing exogenous insulin requirement without evoking an immune response [4,19]. However, such studies presented a limited sample size, therefore, larger studies are still necessary to strengthen the feasibility of using MSCs in clinic.

Finally, although pancreatic islets are always derived from a donor, MSCs can be either of autologous or exogenous origin: these cells are hypoimmunogenic, being lacking of HLA-DR markers, and therefore are theoretically unable to trigger an immune response. However, this is not a unanimous statement, since these cells have been reported to induce an immunogenic response in some cases, which could eventually reduce their effectiveness [47]. In the same way, it must also be underlined that several authors observed that MSCs derived from diabetic patients failed to produce a positive effect, and they could even contribute to disease progression, mainly due to the detrimental effect of the microenvironment they are derived from [48,49]. Therefore, despite the number of authors acknowledging the safe use of allogenic MSCs is larger than those pointing to the contrary; it should be clearly evaluated if allogenic and autologous MSCs share the same potential.

## 4. Conclusions

Despite all the positive evidence, a question still remains clearly unanswered: are MSCs a trump card for the treatment of diabetes? Are they really able to replace the exogenous insulin administration? Probably not in the near future. We are waiting for the results of new and larger clinical trials, but at the moment the unsolved questions of MSCs clinical use outweigh their advantages, overall with respect to insulin administration. Moreover, the analysis of the best time point to perform the MSC administration, alone or co-transplanted with pancreatic islets, is worthy of careful and focused attention. Although many studies took in account patients with a long history of diabetes, considering the overall mechanisms proposed to explain MSCs positive effect, it would be more useful that MSCs were administered as soon as possible, with the aim to counteract the pathogenic mechanisms responsible for diabetes onset. Such a situation is very unlikely to happen for T1D, since the disease becomes evident when most of the pancreatic islets are already lost. This does not preclude that promptly giving MSCs, it would be possible to “save” the function of the residual islets and to promote their regeneration. Moreover, the could contribute to avoiding, or at least to reducing, the long-term side effects of diabetes, that are diabetic nephropathy, neuropathy and retinopathy. Some authors reported a better effect of MSCs in newly-diagnosed patients [50], however, most clinical trials observed good outcomes even in late-onset patients [4]. Before the introduction of MSCs in clinics, it would be necessary, to identify both the best time point from the diagnosis for the transplantation and the worst one, in order to achieve the greatest effect.

Concerning the different kinds of diseases, in particular T1D and T2D, it is necessary to establish if they both respond in a similar way to the MSCs treatment and overall if, in both cases, the treatment is so effective as to replace the insulin administration therapy. Although T2D is a better candidate, as also reported by Cho and colleagues [9], T1D represents the real challenge for researchers. Albeit the MSC treatment could be started just when the islets are already compromised for the most part, the results obtained in the pre-clinical models are really promising, and the improvement of the MSCs differentiation into insulin producing cells, as well as the enhancement of their support to endogenous islet regeneration, could be able to avoid, or at least to reduce, the amount of required insulin, and to allow better and more physiological control of blood glucose levels.

Finally, further detailed analysis and a long-term follow up are necessary to clarify the effect of MSCs manipulation and the tumorigenic effect of some protocols. Despite the aberrant chromosomal alterations observed in cultures [51], so far MSCs have not evidenced a clear tumorigenic potential [52]. Moreover, these cells have a limited expansion rate, different from other stem cell populations. However, it is not negligible that MSCs represent a heterogeneous cellular population, which could include potentially dangerous cells. For these reasons, long-term follow up studies on MSC clinical safety are still necessary, and such a risk should always be considered in a risk-beneficial evaluation. Probably, when a defined protocol with a large consensus is identified and verified, it will be possible to factually hypothesize replacing insulin administration with a new MSCs-based therapy, with or without exogenous pancreatic islets.

## Figures and Tables

**Table 1:** In vivo studies on MSC and diabetes.

Authors	Source of MSCs	Application	Suggested Mechanism
Banerjee et al., 2005 [30]	Mouse BM-MSCs	T1D	Islet regeneration
Domouky et al., 2017 [31]	Rat BM-MSCs	T1D	Islet regeneration
Ezquer et al., 2012 [24]	Mouse BM-MSCs	T1D	
Gao et al., 2018 [33]	Human Wharton’s jelly MSCs	T2D	Immunomodulatory effect
Kuljanin et al., 2017 [22]	Human BM-MSCs	T1D	Immunomodulatory effect
Mahdipour et al., 2019 [34]	Human menstrual blood-derived MSCs	T1D	Islet regeneration
Monfrini et al., 2017 [29]	Rat BM-MSCs	T1D	Islet regeneration
Montanari et al., 2017 [35]	Human BM-MSCs	T1D	Increased Islet function
Nojehdehi et al., 2018 [21]	Mouse Adipose MSCs	T1D	Islet regeneration
Rackham et al., 2013 [36]	Mouse Adipose MSCs	T1D	Increased Islet function
Rackham et al., 2018 [28]	Mouse Adipose MSCs	T1D	Islet regeneration
Sun et al., 2018 [37]	Human umbilical cord-derived MSCs	T2D	Increased Islet function
Xiang et al., 2018 [38]	Mouse BM-MSCs	T1D	Islet regeneration
Xie et al., 2016 [26]	Human umbilical cord-derived MSCs	T2D	Increased Islet function
Yin et al., 2018 [27]	Human umbilical cord-derived MSCs	T2D	Immunomodulatory effect

BM-MSCs: bone marrow-derived MSCs.
